# Increased signal complexity is associated with increased mating success

**DOI:** 10.1098/rsbl.2022.0052

**Published:** 2022-05-18

**Authors:** Noori Choi, Matt Adams, Kasey Fowler-Finn, Elise Knowlton, Malcolm Rosenthal, Aaron Rundus, Roger D. Santer, Dustin Wilgers, Eileen A. Hebets

**Affiliations:** ^1^ School of Biological Sciences, University of Nebraska-Lincoln, Lincoln, NE 68588‐0118, USA; ^2^ Saint Louis University, Saint Louis, MO 63103, USA; ^3^ University of Oklahoma School of Community Medicine, Tulsa, OK 74135, USA; ^4^ University of California-Berkeley, Berkeley, CA 94720, USA; ^5^ West Chester University, West Chester, PA 19383, USA; ^6^ Aberystwyth University, Aberystwyth, Ceredigion, SY23 3DA, UK; ^7^ McPherson College, McPherson, KS 67460, USA

**Keywords:** signal complexity, mate choice, *Schizocosa* wolf spiders, substrate-borne vibratory signals, behavioural plasticity, sexual communication

## Abstract

The evolution of complex signals has often been explored by testing multiple functional hypotheses regarding how independent signal components provide selective benefits to offset the costs of their production. In the present study, we take a different approach by exploring the function of complexity *per se*. We test the hypothesis that increased vibratory signal complexity—based on both proportional and temporal patterning—provides selective benefits to courting male *Schizocosa stridulans* wolf spiders. In support of this hypothesis, all of our quantified metrics of vibratory signal complexity predicted the mating success of male *S. stridulans.* The rate of visual signalling, which is mechanistically tied to vibratory signal production, was also associated with mating success. We additionally found evidence that males can dynamically adjust the complexity of their vibratory signalling. Together, our results suggest that complexity *per se* may be a target of female choice.

## 1. Background

Many animals communicate with complex displays consisting of multiple signals or components within and across sensory modalities [1–6]. Given the costs of producing multiple signal components, such as time/energy loss and increased predation risk [1,7,8], complex signals are hypothesized to have selective benefits like better signal transmission [9–11], increased accuracy in mate assessment [12–14] or increased quantity of information [15–17]. Indeed, there exists a multitude of functional hypotheses regarding why animals engage in complex multi-modal signalling (reviewed in [3]).

To test the potential selective benefits of complex signals, researchers have investigated the functions of complex signals as (i) a simple summing of discrete or overlapping functions of individual signal components [12,14,18,19] or (ii) the emergent properties from the architectures or temporal patterns of multiple signal components [4,14,18–24]. Selection on signal complexity *per se*, however, both within and between sensory modalities, has received less recent attention. Nonetheless, signal complexity has been quantified and studied using various measures—e.g. the number of discrete signals/components [18,25–27], the proportions or the temporal order of signal components [28–31]. Many previous studies have focused on either the function of individual components or the number of signal components/repertoires [14,18,25,26,32], or the function of signal interactions, overall display architecture or temporal order [4,20–24,33–35].

Animals can use a diversity of mechanisms or processes to encode meaning in communication displays [21,36], and thus the integration of multiple approaches is essential to understanding the evolution of complex signals [36,37]. For instance, if a larger courtship signal repertoire reflects better signaller quality (i.e. European starling; [27]), then the quantification of signal complexity only based on the variation in temporal orders may not fully capture the selective benefits of complex signalling. Similarly, when visual ornamentations are presented to receivers through complex behavioural gestures, analysing the diversity of ornamentations alone may only partially elucidate why complexity evolved [38,39]. In the present study, we compare multiple metrics of vibratory signal complexity and signalling rate in a multi-modal signalling wolf spider to test the hypothesis that increased vibratory signal complexity provides males with fitness benefits.

Male *Schizocosa stridulans* wolf spiders produce multi-modal courtship displays consisting of two discrete substrate-borne vibratory components and static/dynamic visual signal components [40–44]. Females are more likely to mate with males that produce more dynamic visual signals (leg-taps) [45], but it has not yet been tested whether the complexity of vibratory signals influences male mating success (additional information in electronic supplementary material, S1). This study investigates potential mechanisms of complex signal evolution by quantifying and comparing male vibratory signal complexity using multiple complexity metrics and determining how they relate to male mating success.

## 2. Material and methods

### (a) Study animals

We collected penultimate (one moult before final maturation) *Schizocosa stridulans* females and males from Marshall Co., MS, USA (34°40′ N 89°28′ W) on May 17–18, 2008. In the laboratory space with a controlled light cycle (12 L : 12 D cycle) and temperature (23 ± 2°C), we housed animals in individual plastic cages (60 × 60 × 80 mm; electronic supplementary material, S1).

### (b) Mate choice assays

We conducted mating trials in a circular, plastic-walled experimental arena (125 mm diameter) within a soundproof chamber (500 × 370 × 430 mm; Super Soundproofing Co., San Marcos, CA, USA). We used filter paper (Whatman^™^; 125 mm radius) as the substrate upon which the spiders courted and from which we recorded vibrations. To elicit male courtship, a mature female *S. stridulans* resided on the filter paper for 1 h before the trial, during which time she deposited pheromone-laden silk on the substrate [46].

During a mating trial, we introduced a female 5 min before a male and then let the female and male interact for 20 min. We recorded male courtship displays with a laser Doppler vibrometer (Polytec PDV-100, Polytec GmbH, Waldbronn, Germany) and a webcam camera (Logitech Webcam Pro 9000, Logitech, Fremont, CA, USA). To increase the signal strength from the vibrometer, we put a piece of reflective tape (5 × 5 mm; 3M Diamond Grade, 3M, Saint Paul, MN, USA) at the centre of the filter paper. The sound and video recordings were encoded into an AVI file on an Apple PowerBook. We used the first 5 min of male courtship for data analysis. We conducted a total of 44 mating trials.

### (c) Quantification of vibratory signal complexity

**Figure 1.**
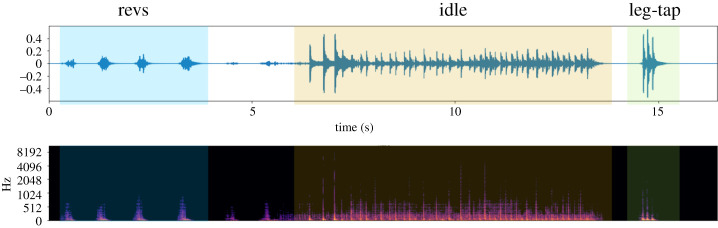
Examples of discrete vibratory signal components by manual classification (revs—blue, idle—orange and leg-tap—green).

To quantify vibratory courtship signal complexity, we converted vibratory signals into a temporal sequence of discrete signal components—rev, idle, and leg-tap [47]—by manual classification (figure 1). We quantified the signal complexity of individual signalling males using three different metrics; (i) Lempel-Ziv complexity [48], (ii) Shannon entropy (hereafter, entropy) [49] and (iii) first-order Markov entropy rate (hereafter, entropy rate) [50] (electronic supplementary material, S2). We tested the underlying assumption of the metrics (electronic supplementary material, S3).

### (d) Quantification of courtship rate

As a more traditional proxy of signal complexity, we calculated (i) *multi-modal* and (ii) *visual-only* courtship rates by dividing the total number of (i) all signal components (vibratory + visual = multi-modal) or (ii) only visual-associated signal components (visual only) by the total courtship duration.

### (e) Statistical analysis

To test the hypothesis that increased vibratory complexity provides males with a reproductive advantage, we constructed binary logistic regression models with each of the vibratory signal complexity metrics as predictor variables and mating success as the response variable. We also used the same binomial logistic regression models with the two proxies of courtship rate as predictor variables—multi-modal and visual-only courtship rates. We used the lm() and glm() in the lme4{} R package [51] and the *p*-values of predictors were calculated using the Anova() in the car{} R package [52]. All statistical tests were conducted using R v. 3.6.1 for Windows [53].

We also investigated the effects of (i) female feedback and (ii) female weight on male vibratory courtship signals (see electronic supplementary material, S4–S6).

All the data and codes for analysis are given in the Dryad Digital Repository [54].

## 3. Results

Among the 44 *Schizocosa stridulans* males, we had nine males that copulated within 25 min (four males within 5 min; five males within an additional 20 min). There was no significant difference between female and male body mass, body mass ratio between females and males, the latency to the first vibratory component, or the number of vibratory components between trials in which males did or did not copulate (table 1).

**Table 1.** Comparison of morphological traits and signal characteristics between non-copulated and copulated trials (mean ± s.d., Welch's *t*-test).

mating	*N*	male body mass (g)	female body mass (g)	body mass ratio (female/male)	number of components	latency (s)
*N*	35	0.041 ± 0.008	0.051 ± 0.010	1.283 ± 0.380	93.9 ± 61.6	89.5 ± 86.1
*Y*	9	0.039 ± 0.007	0.057 ± 0.012	1.489 ± 0.453	103.0 ± 46.7	77.0 ± 83.6
statistical significance		*t =* 0.608, d.f. = 13.866, *p =* 0.553	*t = −*1.454, d.f. = 11.080, *p =* 0.174	*t = −*1.257, d.f. = 11.068, *p =* 0.235	*t = −*0.538, d.f. = 15.998, *p =* 0.598	*t = −*0.422, d.f. = 12.740, *p =* 0.683

**Figure 2.**
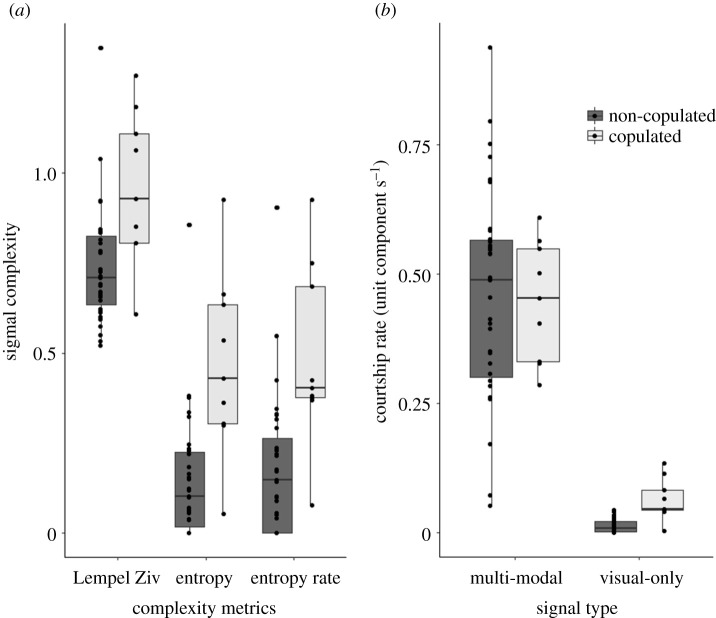
Comparison of (*a*) vibratory signal complexity metrics and (*b*) multi-modal and visual-only courtship rates of non-copulated and copulated males. The top, middle and bottom of the boxes represent the 25th, 50th and 75th percentiles respectively. The whiskers represent 1.5 times the interquartile range or the most extreme value. The dots represent individual data.

Copulated males produced more complex signals (as calculated by each of our complexity metrics) than non-copulated males (table 2 and figure 2). Multi-modal courtship rate was not a significant predictor of male mating success, but the visual-only courtship rate was, with higher visual courtship rates in copulated males (table 2 and figure 2).

**Table 2.** Complexity metrics and multi-modal/visual courtship rate of non-copulated and copulated male *S. stridulans* (mean ± s.d.). Significant predictors of mating success are shown in italics.

mating	normalized Lempel-Ziv	entropy	entropy rate	multi-modal courtship rate	visual courtship rate
*N*	*0.741 ± 0.162*	*0.145 ± 0.169*	*0.174 ± 0.191*	0.459 ± 0.201	*0.013 ± 0.013*
*Y*	*0.959 ± 0.214*	*0.467 ± 0.255*	*0.488 ± 0.255*	0.447 ± 0.117	*0.064 ± 0.040*
statistical significance	*Wald* $x_{1}^{2}=8.663$**,** *p = 0.003*	*Wald* $x_{1}^{2}=13.481$**,** *p < 0.001*	*Wald* $x_{1}^{2}=11.744$**,** *p = 0.001*	Wald $x_{1}^{2}=0.032$, *p* = 0.859	*Wald* $x_{1}^{2}=24.081$**,** *p < 0.001*

## 4. Discussion

Male vibratory courtship complexity, as quantified using proportional (entropy) and temporal patterning (Lempel-Ziv complexity and entropy rate), was associated with mating success of *Schizocosa stridulans* males. Assuming that male mating success is dictated by female mate choice in our experiments, our results are consistent with the hypothesis that vibratory courtship signal complexity itself is under direct selection from female choice. Vibratory signal complexity predicted a male's mating success regardless of which complexity metric, and thus which dimension of signal complexity, we used. This similarity across metrics may be due to the highly stereotyped pattern of signal components in this species. In other words, there is little opportunity for temporal patterns to vary independently of variation in component occurrence in our particular species. Usually, *S. stridulans* males produce 1–2 idle/leg-taps after many revs, and the consecutive occurrence of more than 2 idle/leg-taps is very rare (N. Choi 2020, personal observation; figure 1). Therefore, within *S. stridulans*, the complexity metrics change similarly with differences in the numbers of revs before idle/leg-taps, which vary among or within individuals.

A more traditional analysis using visual-only courtship rate, but not multi-modal courtship rate, was also a significant predictor of male mating success. The component of signalling used to calculate visual courtship—i.e. leg-taps—does, however, occur coincident with the idle vibratory signal component. Thus, more idles necessarily equate to more leg-taps. This fits with the results from simulated sequences (electronic supplementary material, S3), which suggests that the proportion of visual-associated signal components (i.e. idles) may play a major role in the variation in vibratory signal complexity among males. The similarity in findings across our approaches (vibratory complexity versus visual-only courtship rate) raises interesting questions about how these spiders might functionally alter vibratory signalling and whether it is (i) a simple summing of functions of individual signal components [12,14,18,19] or (ii) the emergent complexity from the architectures or temporal patterns that is driving increased likelihood to mate.

We have identified four non-mutually exclusive explanations for the discovered link between our calculated complexity metrics: visual-only courtship rate and male mating success. First, female preferences for other traits may result in a non-causal correlation between male mating success and vibratory signal complexity/visual courtship rates. For example, females may be receptive to males for other reasons unrelated to courtship behaviour (e.g. size and degree of ornamentation) and may provide feedback to these males. In response to such positive feedback, males may increase their courtship rate [55] by integrating more leg-taps (with idles), which necessarily results in increased vibratory signal complexity. Previous studies on *S. stridulans* found an increase in revs and idles immediately before mounting [42,56]. We attempted to test this possibility by comparing male courtship complexity during the first and second halves of our observed courtship window (electronic supplementary material, S4). In support of males altering signal complexity throughout an interaction, we did find that copulated males significantly increased the transition patterns among signal components (Lempel-Ziv complexity and entropy rate) but did not change the proportion of signal components or the number of unit signal components (entropy and multi-modal signal complexity) in the second half of courtships. Additional results of our study similarly demonstrate that (i) male *S. stridulans* can alter the complexity of their vibratory signalling according to female body condition (electronic supplementary material, S6) and (ii) males that produced more complex signals decreased the duration of revs and increased the number of idles (electronic supplementary material, S7). Given these two findings, it seems likely that males alter their signalling complexity and the associated acoustic characters according to female feedback and receptivity [55,57,58].

A hypothesis of female feedback driving increased signal complexity might also predict that positive feedback is tied to male characteristics such as size. In our study, however, we found no difference in body mass between copulated and non-copulated males. Similarly, Rosenthal & Hebets [45] showed that the feeding history of *S. stridulans* males during juvenile or adult life stages did not influence the male size, secondary sexual traits and/or mating success. Thus, the variation in visual courtship rate and signal complexity is likely not facilitated by the difference in male body condition and related female feedback.

Similar to our initial hypothesis that the relationship between vibratory signal complexity and mating success may be the result of female preference and positive feedback on non-signalling traits (e.g. size, degree of ornamentation), this pattern may also be a by-product of the functional variation among isolated vibratory or visual signal components. For example, an increasing proportion of idles/leg-taps necessarily leads to higher calculated complexity metrics. It remains possible that the numbers of idles and/or leg-taps are most important in mating success, not the vibratory signal complexity *per se*. A prior study in *S. stridulans* that explored the role of vibratory versus visual signalling in mating success, however, found no support for a role of visual signalling in the absence of vibratory signalling [11]. Furthermore, we found no evidence that either vibratory complexity or visual courtship rate influenced female orientation behaviour; a behaviour used previously as a proxy of a female's interest in mating [41,59,60] (electronic supplementary material, S5). Copulating males in our study also did not increase the occurrence of particular courtship components (idles/leg-taps), as measured by entropy or courtship rate, in the second half of courtship. Taken together, these results suggest that it is not the visual-vibratory-associated courtship components (idles/leg-taps) that are driving our mating success results, but vibratory signal complexity itself.

In contrast to the by-product explanations, females may prefer more complex male signalling. This may be due to a variety of factors such as increased messaging or content [61,62], a sensory or processing bias [63,64], or preference for complexity *per se* [47,65,66]. Rev, idle and leg-tap signal components, each of which is produced through different production mechanisms [47], may convey different information about male quality. While we do not have evidence to test these hypotheses, previous studies suggested that the larger amount of information in male courtship signals may increase mating success in other species [17,67–69]. In addition, selection exerted through female mate choice might also favour higher signal complexity itself. Such selection could be driven by (i) female preference for high-quality males as indicated by their ability to increase courtship complexity [17,67–69], (ii) sensory biases in females for the structural complexity in male vibratory signals [70,71], or even (iii) aesthetic preferences and/or selection for decreased processing costs [72]. Future work is necessary to tease apart these hypotheses.

In summary, our data provide evidence of direct fitness benefits to male *S. stridulans* that engage in more complex vibratory courtship signalling. Exactly why females are more likely to accept males with more complex displays, however, remains an open question. Nonetheless, despite presumably higher costs of increased signal complexity [7], our data demonstrate that *S. stridulans* males can and will actively alter their signal complexity and that this ability may be under direct selection from females. We anticipate that future work investigating the costs and benefits of complexity *per se* across disparate animal displays, with a special focus on the relationships between modality-specific signalling and overall complexity, will greatly enhance our understanding of how and why many observed animal displays are so complex.

## Data Availability

All data are submitted as electronic supplementary material and uploaded in Dryad. Data and codes for data analysis are given in Dryad Digital Repository: https://doi.org/10.5061/dryad.vt4b8gttk [54]. The data are provided in the electronic supplementary material [73].
